# The Effects of Immersive Virtual Reality in Reducing Public Stigma of Mental Illness in the University Population of Hong Kong: Randomized Controlled Trial

**DOI:** 10.2196/23683

**Published:** 2021-07-14

**Authors:** Anna S Y Yuen, Winnie W S Mak

**Affiliations:** 1 Department of Psychology The Chinese University of Hong Kong Hong Kong Hong Kong

**Keywords:** immersive virtual reality, narrative persuasion, public stigma, mental health stigma, sense of embodiment, story transportation, stigma intervention

## Abstract

**Background:**

Public stigma in mental health often brings various adverse effects on people with mental illness. Researchers have been developing different interventions in combating public stigma.

**Objective:**

This study investigates the effects of immersive virtual reality (IVR) in reducing the public stigma of mental illness using a single-blinded randomized control trial.

**Methods:**

A pre-post experimental design with a 1-week follow-up was conducted. Participants (N=206) were recruited through the mass mail system of The Chinese University of Hong Kong and randomized into 3 conditions: immersive animation, text, and control. In the immersive animation condition (n=72), participants experienced the simulation of daily life and the stigma experienced as an animated story protagonist with mixed anxiety and depressive disorder with IVR. In the text condition (n=65), participants experienced an identical story to the immersive animation condition with first-person audio narration using the same virtual reality headset. In the control condition (n=69), participants watched a video about planets with IVR. All participants received interventions with a researcher-assisted Oculus Go virtual reality headset. Participants’ public stigma was measured through self-administered online questionnaires and compared across conditions and at different time points using repeated measures analysis of variance. Simple and sequential mediation analyses on the relationship of condition (immersive animation vs text) and follow-up public stigma with possible mediators, including sense of embodiment and story transportation, were conducted using PROCESS.

**Results:**

Public stigma did not differ significantly across conditions at pre-experiment (*P*>.99). In the immersive animation and text conditions, public stigma was significantly reduced at postexperiment and at the 1-week follow-up compared to pre-experiment (all with *P*<.001). Public stigma in the control condition at postexperiment and follow-up remained unchanged compared with pre-experiment (*P*=.69). Immersive animation had significantly lower public stigma than the control at postexperiment (*P*=.003) and follow-up (*P*=.02). Text also had lower public stigma than the control at postexperiment (*P*=.007) and follow-up (*P*=.03). However, immersive animation did not significantly differ from text in public stigma at postexperiment and follow-up (both *P*>.99). In simple mediation models, both sense of embodiment (95% CI –0.22 to 0.46) and story transportation (95% CI –0.18 to 0.00) were not significant mediators. In the sequential mediation model, both sense of embodiment and story transportation were significant sequential mediators. Sense of embodiment was positively associated with story transportation (*P*<.001), while story transportation was negatively associated with public stigma (*P*<.001). The indirect effect of the sequential mediation model was significant (95% CI –0.38 to –0.11).

**Conclusions:**

This study provides novel findings and a rigorous comparison in understanding the effects of IVR on public stigma. The findings showed that IVR and text with audio narration performed similarly and significantly in stigma reduction. Sense of embodiment and story transportation were found to be sequentially associated with public stigma reduction.

**Trial Registration:**

Centre for Clinical Research and Biostatistics Clinical Trial Registry CUHK_Ccrb00638; https://www2.ccrb.cuhk.edu.hk/registry/public/632

## Introduction

### Background

One of the biggest challenges that people with mental illness face is psychiatric stigma [1,2]. Stigma refers to a phenomenon in which people are socially discredited as a result of their social identity, health condition, or other characteristics deemed “undesirable” by the dominant group [3]. The public’s stereotypical and negative beliefs, prejudicial emotional reactions, and discriminatory responses toward minority or disadvantaged groups in the society are referred to as public stigma [2,4,5]. People endorsing public stigma toward people with mental illness often make negative but inaccurate assumptions about them, including that people with mental illness are violent, dangerous, weak, and childlike [2,6-10]. People with public stigma also tend to negatively label people with mental illness as “them” and regard them as fundamentally different and undesirable to be part of “us” [2,11].

Public stigma often hampers the recovery and reintegration of people with mental illness into society [9,12]. For example, the public who hold prejudice toward people with mental illness as childlike and having mental illness due to weak character may discriminate against them through supporting coercive treatment [13]. The public who find people with mental illness fearful may call for institutional segregation and avoidance [13]. People with mental illness are often deprived of employment opportunities [5,10,14] or are falsely alleged to be involved in violent crimes [2]. Recognizing the existence of public stigma, some people with mental illness may concur and internalize these public views, leading to self-stigma [2,10,15,16]. The “why try” model has illustrated the complex process of how people with mental illness may be aware of, agree with, and apply the stereotypes of mental illness [17]. As a result, self-esteem and self-efficacy are undermined, which yields “why try” responses in pursuit of life goals [17]. People with mental illness may think they are not worthy of attaining life achievements [17] and have reduced help-seeking behavior [1,5,14,16,17]. Regarding these alarming consequences brought by stigma, varied efforts have been advocated to reduce public stigma and empower people with mental illness.

### Prior Work of Combating Public Stigma

Past efforts in combating public stigma found education and contact between the public and people with mental illness to be the most effective strategies [5,7,18]. Education mainly aims at replacing stigmatizing myths with accurate information and knowledge, and contact attempts to replace negative stereotypes with a positive experience through interpersonal contact on an equal footing [7]. Education and contact significantly led to positive attitudinal change. Among all, contact seemed to yield the most efficacious effect in reducing public stigma, especially for adults [2,5,19].

However, as in vivo contact required presenters to travel to venues and be well trained, it could be tedious and hard to implement widely and frequently [4]. Researchers have therefore investigated the possibilities of using other types of contact [4,20,21]. Filmed contact has been used, and it yielded similar or weaker effects than in vivo contact [4,19-21]. Recently, researchers have begun to investigate the effect of immersive virtual reality (IVR) in reducing public stigma, although this effort is limited [22].

### Reducing Public Stigma With Immersive Virtual Reality

With technological advancement, IVR becomes a possible option to reduce negative stereotypes and prejudice. IVR allows people to be put into another character by changing their body representation [23-25]. It is done by a process referred to as *virtual embodiment*, in which individuals can see a virtual body substituting their bodies through a head-tracked head-mounted display [23,24]. Some can even wear body-tracking suits to induce synchronous virtual body movements [24]. Previous research revealed that IVR could successfully induce ownership over bodies and identities of outgroups and significantly reduce negative stereotypes, including reducing implicit racial bias [24] and stigma toward older adults [26]. The public generally regard people with mental illness as different and outgroup members [2,11]. With IVR, participants could have first-person experiences as people with mental illness. They might regard people with mental illness as ingroup members and have lower stigma. With the aids of immersive experience, participants’ identities and perspectives might also be changed more easily and substantially than nonimmersive means. Therefore, this study applies IVR and compares it to a nonimmersive medium to investigate its effects in reducing public stigma toward mental illness.

In previous IVR research related to mental health public stigma, they mainly focused on the simulation of schizophrenic symptoms [22,27]. Instead of reducing stigma, simulation of symptoms through IVR further increased social distance with people with schizophrenia [22]. Simulation of auditory hallucination without using IVR also yielded similar results [27-29]. In these studies, some comparison groups even reduced greater stigma than the simulation conditions [22,29]. Often, the comparison groups highlighted other components than just symptoms, such as feelings, treatments, difficulties, and accomplishments of a person with mental illness [22,29]. Similarly, in the few virtual reality studies, both immersive and nonimmersive, that had successfully reduced public stigma, the simulation condition was either paired with written empathetic tasks [22] or participants could get information more than symptoms [30]. For example, participants could understand symptoms, biographies of people with mental illness, and false negative beliefs in the virtual reality [30]. They were found to have decreased stereotypes and perceived dangerousness of people with schizophrenia [30].

Therefore, rather than emphasizing symptom simulation, this study took a person-centered approach by simulating the daily life interactions and stigmatized experience of a person with mental illness. We hypothesized that IVR that immersed participants into the everyday living of someone with mental illness could significantly reduce public stigma toward mental illness.

### Hypothesized Mediation Models of Immersive Virtual Reality and Public Stigma

#### Sense of Embodiment as Mediator

To examine how the IVR might affect public stigma, mediation analyses were also conducted. To expand the proposed idea of IVR changing stigma through virtual embodiment, one proposed mediator was sense of embodiment. It refers to the sensations of being inside, controlling, and having ownership over an artificial body [23]. It is possible because our body schema and the concept of the self are labile [31]. Through mapping the physical body to the virtual body, our body schema can be radically altered to the virtual one [32]. As an avatar’s body can embed different social roles or meanings, our social identities may also be altered accordingly [33]. Previous IVR literature on racial bias had suggested that with sense of embodiment, participants embedded new physical and conceptual identities [33]. Other racial groups might become in-group to the participants, leading to generalization of affective processing and positive evaluation of the race and a reduction in racial bias [33]. Thus, this study hypothesized that sense of embodiment could mediate IVR’s relationship with stigma reduction though changing body schema and social identity.

#### Story Transportation as Mediator

Another proposed mediator was story transportation. It describes how much an individual can be immersed in a story [34,35]. With strong story transportation, readers can have their cognition, affection, and attention dominated by the story [34-37]. Story transportation was suggested to have various effects on belief and stigma change [38-43]. It might reduce cognitive and elaborative activities that resist persuasion [42,43]. It could also elicit broad affective responses that allow participants to be more receptive to belief change [42]. People might also have more vivid mental images that make contents and messages more memorable, leading to a greater intention of belief change [42,43]. A previous empirical study had shown that with story transportation, participants felt related to the story protagonist through reading a written vignette and had reduced social distance with people with depression [37]. With various special features of IVR, such as the first-person field of view and tracking of head movements, IVR might result in stronger engagement with the story [44], thus stronger stigma change than a nonimmersive medium.

#### Sense of Embodiment and Story Transportation as Sequential Mediators

Besides testing a sense of embodiment and story transportation as stand-alone mediators, this study also attempted to explore the possible sequential effects of sense of embodiment and story transportation in the same mediation model. In this study, participants would be experiencing the story as the protagonist. Therefore, embodying the avatar could readily enable the participants to become the story protagonist themselves and be part of the virtual settings. Thus, it was hypothesized that sense of embodiment might increase story transportation, leading to reduced public stigma.

## Methods

### Trial Registration

This study was approved by the Survey and Behavioral Research Ethics Committee at The Chinese University of Hong Kong (approval reference number: SBRE-18-078). It is registered at The Centre for Clinical Research and Biostatistics Clinical Trial Registry (trial registration number: CUHK_CCRB00638).

### Recruitment

The study was conducted from January to March 2019. A single-blinded randomized controlled trial was used for this study. According to previous studies that aimed at changing attitudes to outgroup members using IVR, a medium effect size was observed [24]. Meta-analysis showed that video contact and in vivo contact had medium to large effect sizes respectively on changing stigmatizing attitude [19]. With IVR having stronger perceptive stimulation than video contact, but less intense and interactive than in vivo contact, a medium effect size was hypothesized. Assuming a medium effect size (η_ρ_^2^=0.06) with 80% power and a probability of a type I error of 0.05, a total sample size of 141 was needed. Participants were recruited through the mass mail system of The Chinese University of Hong Kong. The purpose of this study and assignment of conditions were concealed until the debriefing session at the end of the entire study. The email and informed consent form only stated that the experiment would be related to IVR. Since the content of the interventions were in Chinese, participants who were 18 years or older and fluent in Chinese were eligible for this study. After completing the pre-experiment questionnaire online, participants who failed to answer attention check questions correctly (eg, “Please choose extremely disagree.”) were excluded to ensure that participants were answering the questions diligently. Participants who failed to provide correct contact information or any available experiment time slots were also excluded. Afterward, each participant was randomly assigned into one of the three conditions: IVR animation (immersive animation), the first-person audio narration of the same story (text), and IVR space control (control). Simple randomization was used with computerized random numbers. Upon signing the consent form online with checkboxes, participants were administered their assigned condition and completed an online postexperiment questionnaire immediately in the experiment room and 1 week after the experiment at home. A total of HK $50 (US $6.44) were given to each participant as a remuneration of time after the accomplishment of the experiment. Responses from participants who completed the postexperiment and follow-up questionnaires were analyzed.

### Experimental Conditions

All experimental conditions were around 10 minutes long. To avoid any placebo effect and to keep other factors constant, all three conditions were conducted with a researcher-assisted virtual reality headset, Oculus Go (Facebook), which could provide 360° head-tracking IVR with corresponding audio and sound effects. With the exception of the researcher assisting with the operation of the IVR headset at the commencement of the experiment, all presentation of experimental content was automatic with minimal technical support from the researcher to minimize researcher bias. All participants received their assigned condition in a dark room while seated on a swivel chair.

**Figure 1:**
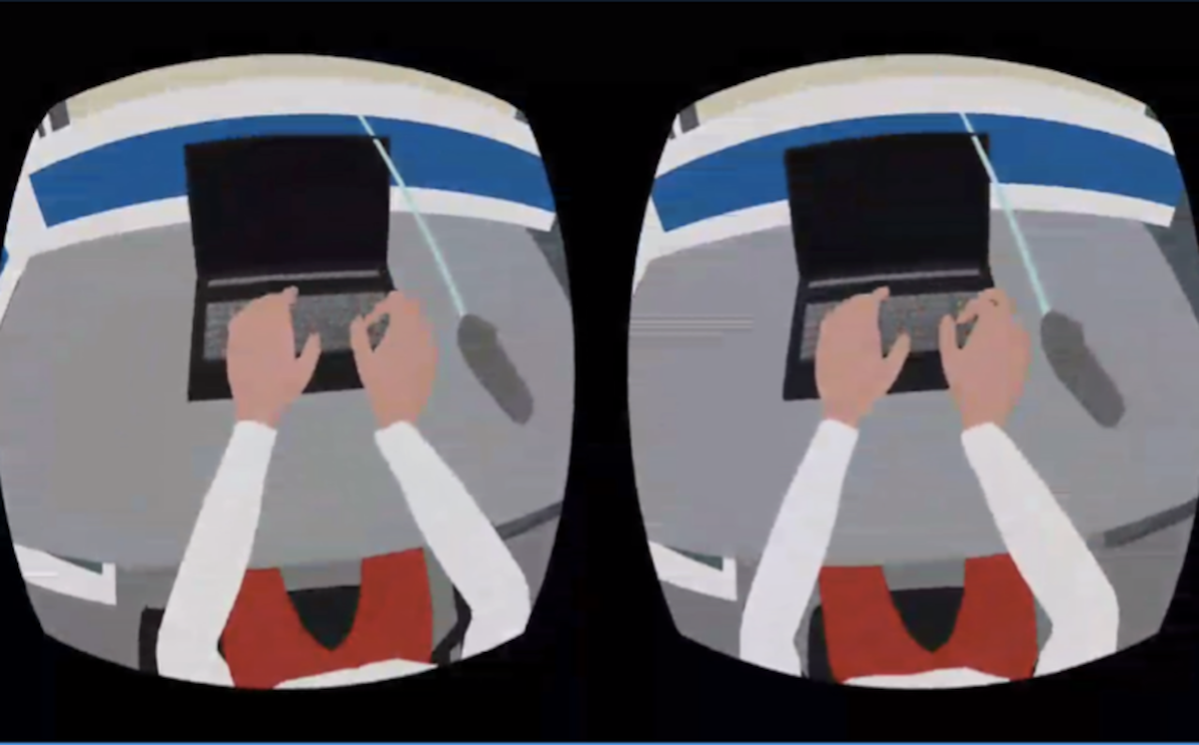
Screenshot of participants seeing the female protagonist’s, Yan’s, body in the immersive animation condition.

**Figure 2:**
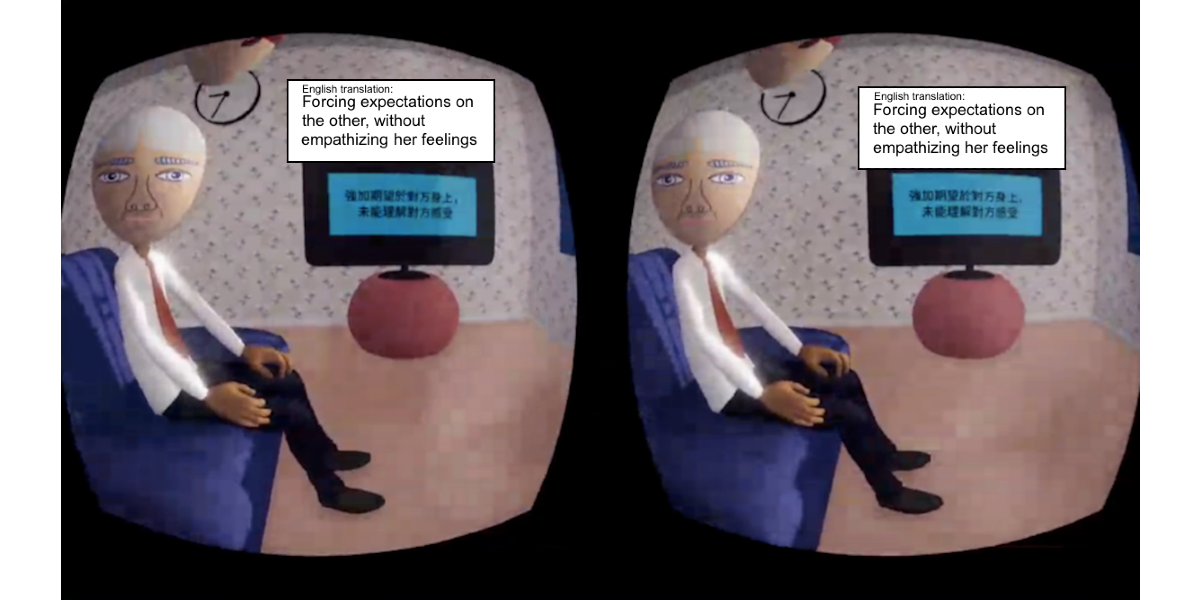
Screenshot of Yan talking to her uncle, and showing how messages pop up to illustrate problematic issues in the uncle’s speech in the immersive animation condition.

In the immersive animation condition, participants were immersed in an animated story as the female protagonist, Yan, who had mixed anxiety and depressive disorder. In the IVR, Yan’s body would be in the position of the participants’ bodies. Participants would be seeing things from Yan’s perspective (Figure 1). The story started with Yan working at the office. The scene then changed to Yan sitting on a sofa alone at home, where stigma from colleagues and pressure from work and family were illustrated. Afterward, Yan talked to her uncle who was visiting. The uncle was an antagonist who stigmatized and imposed more pressure onto Yan. Yan’s field of view would sink while the uncle talked, and everything else would seem larger and higher to create a sense of inferiority. There were also pop-up messages on the side to illustrate the problematic issues in the uncle’s speech during the conversation (Figure 2). Examples included trivializing experience, attributing all responsibilities to the person, and advice-giving without consideration of appropriateness.

**Figure 3:**
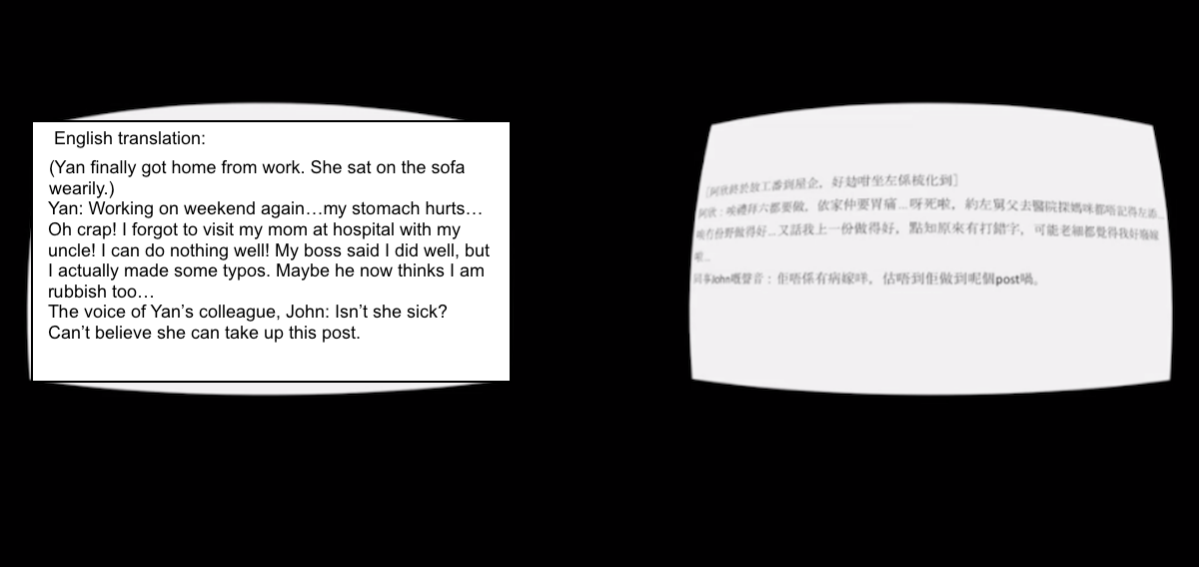
Screenshot of the text condition.

In the text condition, participants read the text and listened with audio narration to the same story as the immersive animation condition. Participants still wore the virtual reality headset to avoid any placebo effect. However, they read the story in a textual and 2D format on a white background with black text (Figure 3). To investigate the separate effects of visual inputs in the IVR condition, the first-person voice-over and sound effects in the IVR remained unchanged here and accompanied the text in accordance to the story development. This condition was a conventional medium that provided a rigorous comparison to the immersive animation condition. It kept the story components identical except for the visual inputs and immersive experience in the IVR. This condition was compared with the immersive animation condition in both the main effects of stigma reduction and mediation analyses. It allowed examination of possible mechanisms of how IVR might affect public stigma differently than nonimmersive means through proposed mediators.

**Figure 4:**
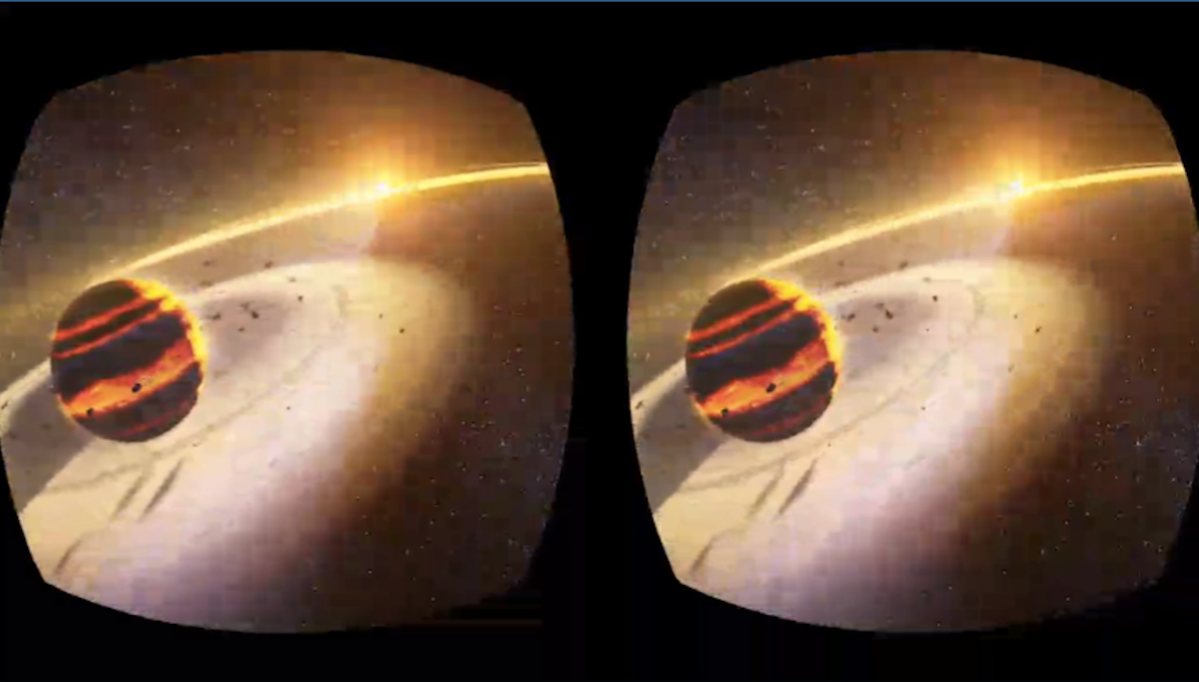
Screenshot of the space control condition.

In the control condition, participants watched a 360° exoplanet virtual reality video with corresponding audio and sound effects. The exoplanet video was titled “Take a Virtual Reality tour of six REAL exoplanets” on YouTube with its copyright owned by the University of Exeter, 2018 (Figure 4). The video was created by the University of Exeter Astrophysics group in partnership with We the Curious and Engine House VFX. This video was used in the study by permission of the University of Exeter.

### Measures

#### The Main Effect on Public Stigma

To measure changes in public stigma, the 21-item Public Stigma and Acceptance Scale [45] was used at pre-experiment, postexperiment, and 1-week follow-up. It measures public stigma and acceptance toward mental illness on a 6-point Likert scale from 1 (strongly disagree) to 6 (strongly agree). It consists of two subscales, 12 items measuring public stigma (eg, “People with mental illness are a burden to the society.”) and 9 items measuring personal advocacy toward accepting people with mental illness (eg, “I take the initiative to reach out to people with mental illness.”). The higher the mean scores of the public stigma subscale and the lower the mean scores of the personal advocacy subscale, the greater the stigma and the lower the acceptance toward mental illness. For the total mean scores, items in the personal advocacy subscale were reverse coded so that higher total mean scores indicated greater stigmatizing attitudes. In this study, total mean scores of all items were used to measure public stigma. Possible total mean scores ranged from 1 to 6. The Cronbach α of the public stigma and personal advocacy subscales were .92 and .87, respectively. The overall Cronbach α was .93 for the entire scale in this study.

#### Mediators

##### Sense of Embodiment

To account for possible mediation effects, sense of embodiment and story transportation were measured post experiment in the immersive animation and text conditions. Since the topic of interest was on what might influence change in public stigma and how IVR’s effect differed from the conventional audio narration, scales on mediation were not administered in the control condition.

Although previous studies have measured sense of embodiment [24,46,47], the items were mostly context-specific to the virtual environments and avatars in those studies. Therefore, with no standardized scale available, a 7-item Sense of Embodiment Scale was developed to fit this experiment context based on previous virtual reality studies [24,46,47] and rubber hand illusion literature [48]. Seven items were developed based on components that were commonly shared among the previous literature, such as ownership (eg, “I felt like I was looking at my own body when I looked at Yan’s.”) and agency (eg, “If I had wanted, I felt like I could have controlled Yan’s body.”). Items were rated on a 7-point Likert scale from 1 (strongly disagree) to 7 (strongly agree; see Textbox 1 for complete scale items). The higher mean score indicated a greater sense of embodiment. The Sense of Embodiment Scale had a Cronbach α of .85 in this study.

**Items in the Sense of Embodiment Scale, with items 6 and 7 as reversed items.**

1. I felt as if I was looking at my own body when I looked at Yan’s.
2. When Yan was sitting on the sofa, I felt as if I was sitting on the sofa.
3. I felt as if Yan’s body was my body.
4. When the uncle was talking to Yan, I felt as if he was talking to me.
5. If I had wanted, I felt like I could have controlled Yan’s body.
6. I found it difficult to get into the role of Yan.
7. I felt as if I was an outsider to the story.

##### Story Transportation

The Transportation Scale [36] measures how well an individual is absorbed into a story. Some wordings of the 15-item scale were modified to fit the current context (eg, changing “While reading the narrative I had a vivid image of Katie” to “While experiencing the story I had a vivid image of Yan.”). The scale was rated on a 7-point Likert scale from 1 (strongly disagree) to 7 (strongly agree). The higher mean score indicated greater devotion in the story. The Transportation Scale had a Cronbach α of .79 in this study.

The Public Stigma and Acceptance Scale and the Sense of Embodiment Scale were originally developed in Chinese. The Transportation Scale was translated into Chinese and back-translated into English to check on conceptual equivalence.

### Data Analysis

Analyses were conducted using SPSS 24.0 for Mac (IBM Corp). First, descriptive statistics were obtained. Information related to age, gender, education, occupation, sexual orientation, religious belief, marital status, mental illness history, and previous social contact with people with mixed anxiety and depressive disorder was obtained. Chi-square tests on nominal demographic variables and one-way analysis of variance (ANOVA) on continuous variables were conducted to examine any systematic differences on demographics among the 3 groups. If there were any systematic differences among the groups, results would be adjusted, controlling for possible confounders. Second, to compare the change in public stigma, repeated measures ANOVA was used. The main effect of groups (immersive animation vs text vs control), time (pre-experiment, postexperiment, follow-up), and their interaction effect (time × group) were examined. The level of statistical significance was set at a *P*≤.05 threshold (two-tailed). If a significant effect was found, post hoc analysis was conducted. Bonferroni-adjusted pairwise comparisons were used to avoid inflated type I error. Third, to test the proposed mediation models, Pearson correlations of condition (immersive animation vs text), sense of embodiment, story transportation, and follow-up public stigma were obtained. A simple mediation analysis was conducted with the condition (immersive animation vs text), sense of embodiment, and follow-up public stigma. A similar simple mediation analysis was also conducted using story transportation as the mediator. To further investigate the relationship of sense of embodiment and story transportation, sequential mediation analysis was also conducted. The condition (immersive animation vs text) and follow-up public stigma were put into the model, having sense of embodiment and story transportation as sequential mediators. The level of statistical significance was set at *P*≤.05 threshold (two-tailed) and determined by 95% CIs. PROCESS on SPSS was used to generate all the mediation analyses. Model 4 was used to generate simple mediation analyses, and model 6 was used to generate sequential mediation analysis according to the suggestion of Hayes [49].

## Results

### Participants

**Figure 5:**
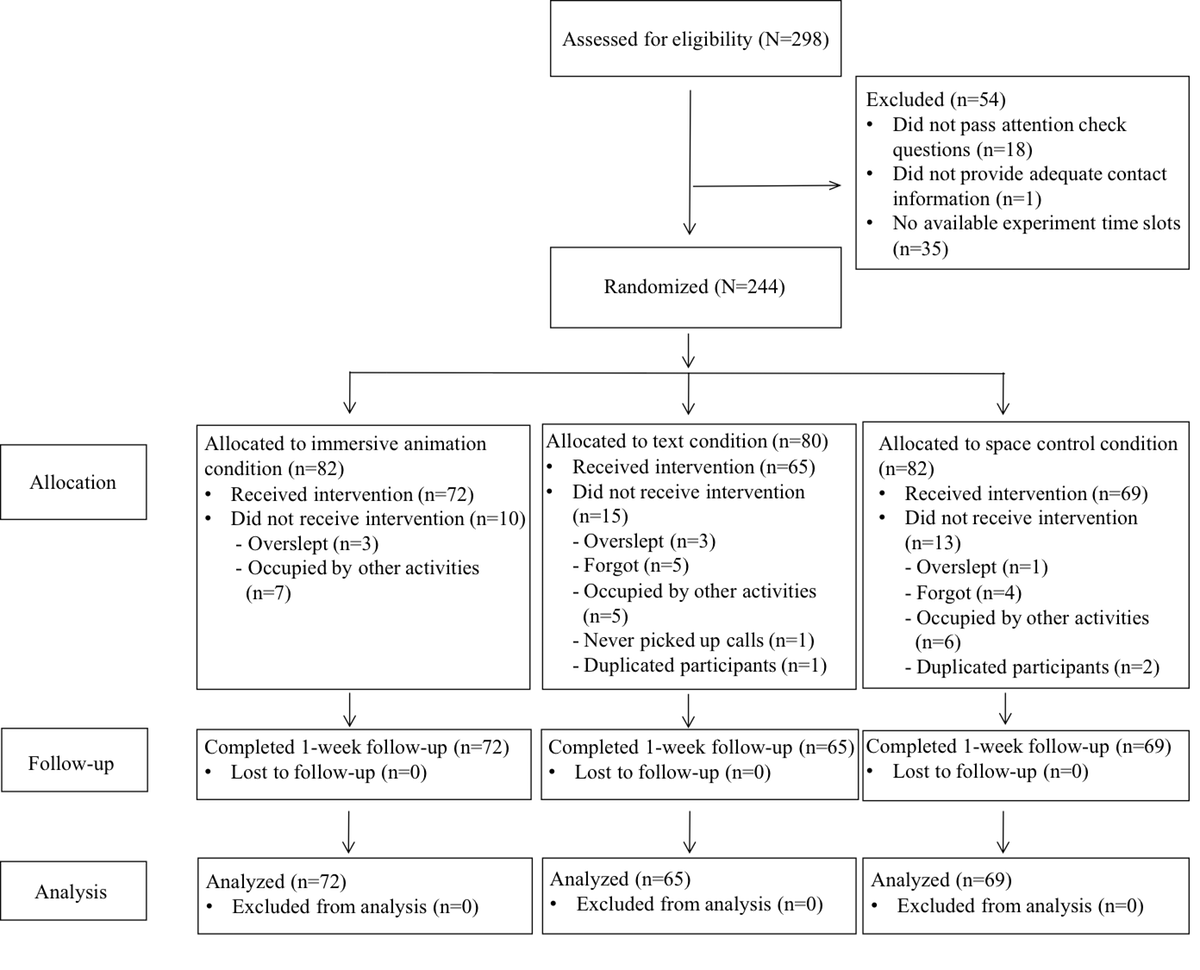
CONSORT (Consolidated Standards of Reporting Trials) diagram of participant recruitment.

Data from 206 participants were analyzed. Out of the 298 participants who provided informed consent and completed the pre-experiment questionnaire, 18 participants who failed to answer the attention check questions correctly, 1 participant who failed to provide adequate contact information, and 35 participants who failed to provide available experiment time slots were excluded (Figure 5). A total of 244 participants were randomized into the three conditions. A total of 35 participants who did not show up and 3 participants who applied to the same experiment twice were excluded. A total of 206 participants went through the experiment and completed questionnaires post experiment and at a 1-week follow-up. The final sample size was 72 for the immersive animation condition, 65 for the text condition, and 69 for the control condition, with a total sample size of 206.

For demographic information, see Table 1. The mean age of participants was 21.76 (SD 5.04, range 18-64) years. Out of the 206 participants, 114 (55.3%) were females and 91 (44.2%) were males. Most participants had a Bachelor’s degree (n=166, 80.6%) and were single (n=198, 96.1%), students (n=173, 84.0%), and heterosexual (n=183, 88.8%). Most of them had no mental illness history (n=198, 96.1%) and no religious belief (n=153, 74.3%). Among the 206 participants, 92 (44.7%) had social contact with people with mixed anxiety and depressive disorder before. Chi-square tests on demographic variables and one-way ANOVA on continuous variables were conducted. No systematic differences in any demographic variables among the three conditions were found. Demographics variables were not expected to impact the results in this study.

**Table 1:** Participant demographic data.^a^

Characteristic			Total (N=206)	Immersive animation (n=72)	Text (n=65)	Control (n=69)	*P* value difference across conditions	
Age (years), mean (SD)			21.76 (5.04)	21.57 (4.18)	21.95 (6.31)	21.77 (4.55)	.91	
**Gender, n (%)**								.68
	Female		114 (55.3)	38 (52.8)	35 (53.8)	41 (59.4)		
	Male		91 (44.2)	33 (45.8)	30 (46.2)	28 (40.6)		
	Others		1 (0.5)	1 (1.4)	0 (0.0)	0 (0.0)		
**Education, n (%)**								.37
	High school		6 (2.9)	1 (1.4)	1 (1.5)	4 (5.8)		
	Bachelor’s degree		166 (80.6)	60 (83.3)	54 (83.1)	52 (75.4)		
	Master’s degree		27 (13.1)	10 (13.9)	7 (10.8)	10 (14.5)		
	PhD		2 (1.0)	1 (1.4)	0 (0.0)	1 (1.4)		
	Others		5 (2.4)	0 (0.0)	3 (4.6)	2 (2.9)		
**Faculty, n (%)**								.93
	Arts		25 (12.1)	9 (12.5)	8 (12.3)	8 (11.6)		
	Business		51 (24.8)	17 (23.6)	17 (26.2)	17 (24.6)		
	Education		14 (6.8)	7 (9.7)	4 (6.2)	3 (4.3)		
	Engineering		16 (7.8)	5 (6.9)	5 (7.7)	6 (8.7)		
	Law		2 (1.0)	0 (0.0)	1 (1.5)	1 (1.4)		
	Medicine		25 (12.1)	5 (6.9)	11 (16.9)	9 (13.0)		
	Science		27 (13.1)	12 (16.7)	6 (9.2)	9 (13.0)		
	Social science		44 (21.4)	16 (22.2)	12 (18.5)	16 (23.2)		
	Others		2 (1.0)	1 (1.4)	1 (1.5)	0 (0.0)		
**Occupation, n (%)**								.29
	Student		173 (84.0)	63 (87.5)	57 (87.7)	53 (76.8)		
	Full-time employment		29 (14.1)	8 (11.1)	7 (10.8)	14 (20.3)		
	Others		4 (1.9)	1 (1.4)	1 (1.5)	2 (2.9)		
**Sexual orientation, n (%)**								.68
	Heterosexual		183 (88.8)	66 (91.7)	58 (89.2)	59 (85.5)		
	Homosexual		11 (5.3)	3 (4.2)	4 (6.2)	4 (5.8)		
	Bisexual		7 (3.4)	2 (2.8)	2 (3.1)	3 (4.3)		
	Others		5 (2.4)	1 (1.4)	1 (1.5)	3 (4.3)		
**Religious belief, n (%)**								.15
		No religious belief	153 (74.3)	47 (65.3)	53 (81.5)	53 (76.8)		
		Christian	43 (20.9)	22 (30.6)	9 (13.8)	12 (17.4)		
		Others	10 (4.9)	3 (4.2)	3 (4.6)	4 (5.8)		
**Marital status, n (%)**								.42
		Single	198 (96.1)	68 (94.4)	62 (95.4)	68 (98.6)		
		Married	8 (3.9)	4 (5.6)	3 (4.6)	1 (1.4)		
**Mental illness history, n (%)**								.46
		Yes	8 (3.9)	4 (5.6)	1 (1.5)	3 (4.3)		
		No	198 (96.1)	68 (94.4)	64 (98.5)	66 (95.7)		
**Social contact with people with MADD^b^, n (%)**								.64
		Yes	92 (44.7)	29 (40.3)	31 (47.7)	32 (46.4)		
		No	114 (55.3)	43 (59.7)	34 (52.3)	37 (53.6)		

^a^All percentages may not total to 100% due to rounding.

^b^MADD: mixed anxiety and depressive disorder.

### Changes in Public Stigma

Repeated measures ANOVA on public stigma yielded a significant interaction (time × group) effect (*F*_4,404_=5.2; *P*<.001; η_ρ_^2^=0.05). The effect size was small. Bonferroni-adjusted pairwise comparisons showed that the immersive animation (mean 2.73, SD 0.64), text (mean 2.78, SD 0.68), and control (mean 2.84, SD 0.64) conditions did not differ in public stigma pre-experiment (*P*>.99). Post experiment, the immersive animation group (mean 2.45, SD 0.63) had significantly lower public stigma than the control group (mean 2.80, SD 0.63; *P*=.003). The text group (mean 2.47, SD 0.62) yielded similar results by having significantly lower public stigma than the control group (*P*=.007). A similar pattern of results was also observed at the 1-week follow-up. The immersive animation group (mean 2.52, SD 0.66) had significantly lower public stigma than the control group (mean 2.82, SD 0.61; *P*=.02). The text group (mean 2.53, SD 0.68) also had significantly lower public stigma than the control group at follow-up (*P*=.03). However, the immersive animation and text groups did not differ from each other significantly at postexperiment and at the 1-week follow-up (*P*>.99; see Table 2 for descriptive statistics and Table 3 for detailed pairwise comparison).

**Table 2:** Public stigma scores across conditions at different time points.

Time points	Immersive animation (n=72), mean (SD)	Text (n=65), mean (SD)	Control (n=69), mean (SD)
Pre-experiment	2.73 (0.64)	2.78 (0.68)	2.84 (0.64)
Postexperiment	2.45 (0.63)	2.47 (0.62)	2.80 (0.63)
Follow-up	2.52 (0.66)	2.53 (0.68)	2.82 (0.61)

**Table 3:** Detailed Bonferroni-adjusted pairwise comparison of public stigma between conditions at different time points.

Time points	Condition	Condition for comparison	Mean difference (SE)	*P* value
Pre-experiment	Immersive animation	Text	–0.05 (0.11)	>.99
Pre-experiment	Immersive animation	Control	–0.11 (0.11)	.99
Pre-experiment	Text	Control	–0.06 (0.11)	>.99
Postexperiment	Immersive animation	Text	–0.02 (0.11)	>.99
Postexperiment	Immersive animation	Control	–0.36 (0.11)	.003
Postexperiment	Text	Control	–0.33 (0.11)	.007
Follow-up	Immersive animation	Text	–0.01 (0.11)	>.99
Follow-up	Immersive animation	Control	–0.30 (0.11)	.02
Follow-up	Text	Control	–0.29 (0.11)	.03

In the immersive animation group, a significant time effect was found (*F*_2,202_=20.0; *P*<.001; η_ρ_^2^=0.165). The effect size was large. The immersive animation group had significantly lower public stigma at postexperiment (mean 2.45, SD 0.63) and follow-up (mean 2.52, SD 0.66) than pre-experiment (mean 2.73, SD 0.64; both with *P*<.001). Public stigma did not significantly differ between postexperiment and follow-up (*P*=.19), implying that the effect at postexperiment was sustained at follow-up for the immersive animation group. In the text group, a significant time effect was also found (*F*_2,202_=21.3; *P*<.001; η_ρ_^2^=0.174). The effect size was large. The text group followed a similar pattern of time effect as the immersive animation group. The text group had significantly lower public stigma at postexperiment (mean 2.47, SD 0.62) and follow-up (mean 2.53, SD 0.68) than pre-experiment (mean 2.78, SD 0.68; both with *P*<.001). No significant difference between postexperiment and follow-up was found for the text group (*P*=.51). In the control group, no significant time effect was found (*F*_2,202_=0.37; *P*=.69; η_ρ_^2^=0.004), and the mean score was 2.84 (SD 0.64) at pre-experiment, 2.80 (SD 0.63) at postexperiment, and 2.82 (SD 0.61) at follow-up.

### Mediation Analyses

The mean scores of sense of embodiment, story transportation, and follow-up public stigma are reported in Table 4. Pearson correlations of variables included in the mediation analyses, including condition (immersive animation vs text), sense of embodiment, story transportation, and follow-up public stigma are also reported in Table 4.

**Table 4:** Correlations and descriptive statistics of variables included in the mediation analyses.

Variables			Correlations of variables included in the mediation analyses						
			Condition (immersive animation vs text)		Sense of embodiment		Story transportation		Follow-up public stigma
**Condition (immersive animation vs text)^a^**									
	*r*	1		0.49		0.17		–0.004	
	*P* value	—^b^		<.001		.051		>.99	
**Sense of embodiment**									
	*r*	0.49		1		0.512		–0.091	
	*P* value	<.001		—		<.001		.29	
**Story transportation**									
	*r*	0.17		0.512		1		–0.354	
	*P* value	.051		<.001		—		<.001	
**Follow-up public stigma**									
	*r*	–0.004		–0.091		–0.354		1	
	*P* value	>.99		.29		<.001		—	
Overall, n			137		137		137		137
Overall, mean (SD)			—^c^		4.25 (1.10)		5.00 (0.66)		2.52 (0.67)
Immersive animation condition, mean (SD)			—^c^		4.75 (0.95)		5.11 (0.59)		2.52 (0.66)
Text condition, mean (SD)			—^c^		3.69 (0.99)		4.89 (0.71)		2.53 (0.68)

^a^Condition was dummy coded with text condition as 0 and immersive animation as 1 in the correlation and mediation analysis. Control condition was not included in the mediation analysis.

^b^Not applicable.

^c^Condition was a dichotomous variable. Mean and SD were not applicable.

**Figure 6:**
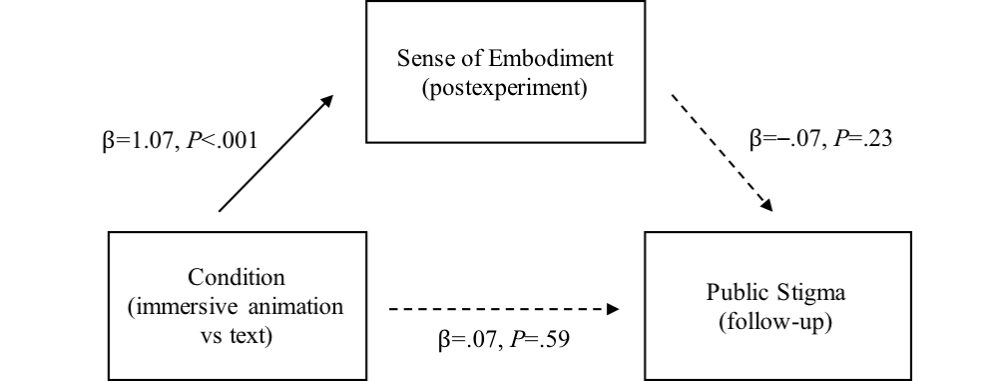
Simple mediation model of the condition, sense of embodiment, and public stigma.

Consistent with the correlation results (Table 4), the simple mediation analysis showed that sense of embodiment was significantly associated with condition (β=1.07; t_135_=6.45; *P*<.001; Figure 6). However, sense of embodiment was not significantly associated with public stigma (β=–.07; t_135_=–1.19; *P*=.23). Therefore, the indirect effect was not significant (95% CI –0.22 to 0.46). The direct effect from condition to public stigma was also not significant (95% CI –0.18 to 0.33).

**Figure 7:**
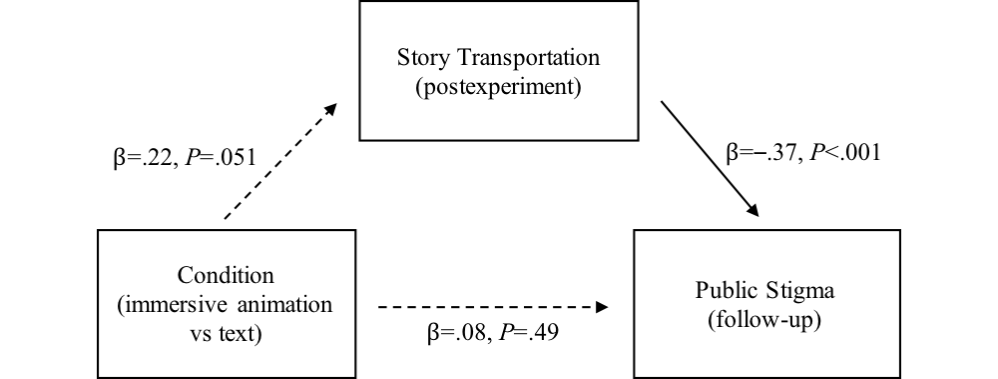
Simple mediation model of the condition, story transportation, and public stigma.

For story transportation, the result was also consistent with the correlation analysis (Table 4). The simple mediation analysis showed that the condition was not significantly associated with story transportation (β=.22; t_135_=1.97; *P*=.051; Figure 7). However, story transportation was significantly and negatively associated with public stigma (β=–.37; t_135_=–4.44; *P*<.001). The indirect effect of condition to story transportation to public stigma was not significant (95% CI –0.18 to 0.00). The direct effect from condition to public stigma was also not significant (95% CI –0.14 to 0.29).

**Figure 8:**
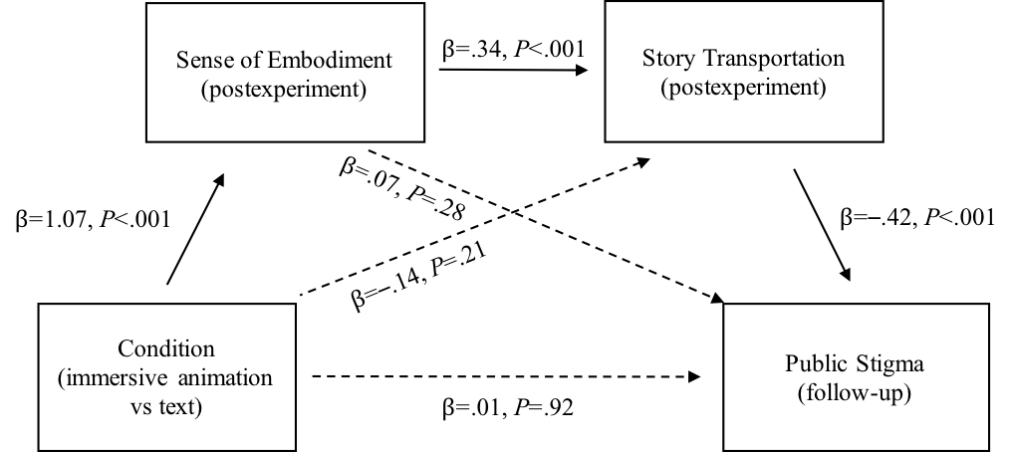
Sequential mediation model of the condition, sense of embodiment, story transportation, and public stigma.

Sense of embodiment and story transportation were then put into the same sequential mediation analysis to investigate their relationship on follow-up public stigma. From the sequential mediation analysis (Figure 8), the relationship of conditions and follow-up public stigma was significantly and sequentially mediated by sense of embodiment and story transportation as hypothesized. In this sequential mediation model, the condition was significantly associated with sense of embodiment (β=1.07; t_134_=6.45; *P*=<.001) but not directly with story transportation (β=–.14; t_134_=–1.26; *P*=.21). Sense of embodiment was significantly associated with story transportation (β=.34; t_134_=6.68; *P*<.001) but was not significantly associated with public stigma (β=.07; t_134_=1.09; *P*=.28). For story transportation, it was significantly and negatively associated with public stigma (β=–.42; t_134_=–4.40; *P*<.001). The indirect effect of condition to sense of embodiment then to story transportation and finally to follow-up public stigma was significant (Figure 8). The standardized indirect effect was –0.15. The bootstrapped unstandardized indirect effect was also –0.15 (95% CI –0.38 to –0.11). However, the direct effect from condition to follow-up public stigma was not significant (95% CI –0.23 to 0.26).

## Discussion

### Overall Effect of Immersive Animation on Public Stigma

This study compared the effect of IVR and text with audio narration in reducing public stigma. Both IVR and text with audio narration successfully reduced public stigma, and the effect was sustained at the 1-week follow-up. In contrast, the control space video did not elicit any change in public stigma as expected. It could be inferred that the simulation of daily interactions and stigma experienced by a person with mental illness could effectively reduce public stigma. The result also supported the hypothesis that IVR could successfully reduce public stigma.

The results were consistent with previous studies that used IVR to reduce negative stereotypes toward outgroup members, including other racial groups [24] and older adults [26]. In the mental health setting, it was also consistent with virtual reality literature that focused on interacting with people with mental illness and dismissing negative beliefs [30]. However, the results in this study were not consistent with previous IVR and studies that simulated symptoms of people with schizophrenia. The simulations of hallucinations had led to increases in social distance and negative attitudes toward people with schizophrenia [22,29]. The different results from this study might be due to the different emphasis of immersive content.

This study had purposely included little simulations of symptoms in the IVR. It aimed to construct the everyday living experience of the story protagonist as a person, instead of just a combination of symptoms. Although, unlike IVR studies on racial groups or older adults, there was not a change in appearance according to an outgroup member in this study, the first-person field of view and immersion as the story protagonist with mixed anxiety and depressive disorder might already have allowed participants to take on the outgroup identity and perspective as a person with mental illness [24]. On the contrary, in previous literature on stigma change toward people with schizophrenia, the simulation of a hallucination might have emphasized the existence of positive symptoms, instead of allowing participants to understand and feel other aspects of the living experience. Positive symptoms that could be perceived as uncontrollable and dangerous by the public might deter participants from engaging with people with schizophrenia [50]. Therefore, simulating positive symptoms might increase social distance and discrimination instead.

On the other hand, different from the hypothesis, IVR did not outperform text with audio narration in reducing public stigma significantly. In the few stigma studies that compared virtual reality with a rigorous comparison group, it was shown that only virtual reality with interactive components such as allowing participants to move closer to other virtual characters had significantly reduced greater stigma than the written vignettes of people with mental illness [51]. Immersive experience without active interaction did not outperform the written vignettes in stigma reduction [22]. Therefore, the visual immersive experience of IVR might not be strong enough to induce a significant difference than text with audio narration in stigma reduction. Interactive features in IVR, such as making choices or controlling objects in the virtual environment [52], or even having body or arm tracking for synchronized movements with avatars, might induce a stronger effect in stigma reduction than text. These features of IVR should be tested in the future.

Another possible explanation of the similar performance of the IVR and text conditions might be due to the type of graphics used. This IVR used animated cartoons. In the text condition, although without pictorial inputs, voice-over and detailed descriptions of the story, including the virtual environment, characters, and actions, were included. It might help with imagining the story in participants’ minds. Those imaginations might perform similarly with animated cartoons in engaging the participants. Thus, having similar effects on stigma reduction. With more realistic animation, IVR might allow participants to resonate better and be more involved in the story [52]. Therefore, IVR with more realistic graphics should be investigated in the future.

### Sense of Embodiment, Story Transportation, and Public Stigma

Moving on to the mediation analyses, although the condition was not directly associated with story transportation, story transportation was found to be a preceding variable directly associated with a reduction in public stigma. As mentioned previously, story transportation emphasized the cognitive and affective immersion in a story [34,36], which might elicit various cognitive and affective effects on belief change [40-43]. It might also reduce elaborative activities that resist stigma change [42,43] and elicit broad affective responses that allow participants to be more conducive to reduce their public stigma [42]. Therefore, stigma change might benefit from the broad cognitive and affective influences by story transportation.

On the other hand, although the condition was associated with sense of embodiment, sense of embodiment was not directly associated with stigma change. However, with the sequential mediation model, sense of embodiment was significantly and positively associated with story transportation, which was then linked to the reduction of public stigma. This showed that the physical sensation of being inside an avatar’s body might not be sufficient for reducing public stigma. However, sense of embodiment might allow participants to feel as if they were the story protagonist and be transported in the story. Afterward, participants might have different cognitive and affective reactions from story transportation, which facilitated reduced public stigma.

The results from mediation analyses also gave more clues as to why IVR and text conditions performed similarly. The only difference between the two conditions was the absence of IVR’s visual inputs and 3D immersive experience in the text condition. However, from the mediation analyses, the intense absorption of thoughts and emotions to the story was more important and closely linked with reducing public stigma than merely the physical sensation in another body. The complexity of stigma change called for more holistic simulation of experiences that allow participants to be more absorbed in the stories, lives, and feelings of a person with mental illness. Thus, the differences in visual inputs or physical experience in this IVR setting might not be sufficient to induce stronger story transportation and stigma reduction than the text condition.

### Limitations and Future Directions

Although this study investigated a novel medium, IVR and its effects on public stigma, several limitations were acknowledged. First, some scales in this study, such as the Sense of Embodiment Scale, were originally developed in this study due to the lack of available scales. Further validation of the newly developed scales is needed. Second, the participants in this study were mainly college students. It might pose a challenge to the generalization of results to the general population. Therefore, replication of the study on a more diverse sample is needed to understand the effects of IVR on the public. Third, moderate to long-term effects of stigma change were unknown. Although there was a 1-week follow-up in this study, the follow-up would still be considered as measuring a rather short-term effect of the interventions [5]. Longer follow-up, such as 1 month, is needed to examine how stigma change may be sustained over time.

Fourth, actual behavioral consequences were not measured. Research showed that there could be a difference between intention and actual behavior [53-55]. Future IVR studies could consider measuring participants’ gaze orientation and interpersonal distance toward a person with mental illness in the virtual environment [56]. This would require participants to walk around the experiment room with full-body tracking [56]. Fifth, compared to text, IVR could be quite novel to most people. Interventions in this study all lasted 10 minutes. Participants might struggle to get accustomed to IVR within a short period. Future studies can give participants an embodiment phase or a practice session to freely familiarize with and explore their virtual bodies and the virtual environment [24,26]. Sixth, in the text condition, there were no pictorial or animated inputs. Filmed contact or videos are of greater visual similarity with IVR than text. In vivo contact even allows participants to have real life perception and interaction with people. They may allow more rigorous comparison with IVR. However, due to the lack of available presenters and funding, in vivo contact, filmed contact, and video were not available in this study. Future studies can consider including them as comparison groups.

Notwithstanding these limitations, this study was one of the few attempts to investigate the effects of IVR on public stigma by comparing it with two rigorously designed comparison groups that attempted to rule out the separate effects of IVR and text with audio narration.

### Conclusion

This study provided novel findings and a rigorous comparison in understanding the effects of IVR as a medium of public stigma reduction. The findings showed that IVR and text with audio narration both performed similarly well in reducing public stigma. Sense of embodiment and story transportation were found to be sequentially associated with public stigma reduction. This study gave a more nuanced understanding to the complexity of reducing public stigma and components in building an effective medium of stigma reduction in the future.

## Appendix

Multimedia Appendix 1 CONSORT-eHEALTH checklist (V 1.6.1).

## Abbreviations

ANOVA: analysis of variance
IVR: immersive virtual reality

## References

[1] Kosyluk KA, Al-Khouja M, Bink A, Buchholz B, Ellefson S, Fokuo K, Goldberg D, Kraus D, Leon A, Michaels P, Powell K, Schmidt A, Corrigan PW (2016). Challenging the stigma of mental illness among college students. J Adolesc Health.

[2] Rüsch N, Angermeyer MC, Corrigan PW (2005). Mental illness stigma: concepts, consequences, and initiatives to reduce stigma. Eur Psychiatry.

[3] Goffman E (1986). Stigma: Notes on the Management of Spoiled Identity.

[4] Chan JYN, Mak WWS, Law LSC (2009). Combining education and video-based contact to reduce stigma of mental illness: "The Same or Not the Same" anti-stigma program for secondary schools in Hong Kong. Soc Sci Med.

[5] Thornicroft G, Mehta N, Clement S, Evans-Lacko S, Doherty M, Rose D, Koschorke M, Shidhaye R, O'Reilly C, Henderson C (2016). Evidence for effective interventions to reduce mental-health-related stigma and discrimination. Lancet.

[6] Crisp AH, Gelder MG, Rix S, Meltzer HI, Rowlands OJ (2000). Stigmatisation of people with mental illnesses. Br J Psychiatry.

[7] Corrigan PW, Green A, Lundin R, Kubiak MA, Penn DL (2001). Familiarity with and social distance from people who have serious mental illness. Psychiatr Serv.

[8] Link BG, Phelan JC, Bresnahan M, Stueve A, Pescosolido BA (1999). Public conceptions of mental illness: labels, causes, dangerousness, and social distance. Am J Public Health.

[9] Pinfold V, Toulmin H, Thornicroft G, Huxley P, Farmer P, Graham T (2003). Reducing psychiatric stigma and discrimination: evaluation of educational interventions in UK secondary schools. Br J Psychiatry.

[10] Seeman N, Tang S, Brown AD, Ing A (2016). World survey of mental illness stigma. J Affect Disord.

[11] Link BG, Phelan JC (2001). Conceptualizing stigma. Annu Rev Sociol.

[12] Wahl OF (2012). Stigma as a barrier to recovery from mental illness. Trends Cogn Sci.

[13] Corrigan PW, Watson AC (2002). Understanding the impact of stigma on people with mental illness. World Psychiatry.

[14] Wahl OF (1999). Mental health consumers' experience of stigma. Schizophr Bull.

[15] Livingston JD, Boyd JE (2010). Correlates and consequences of internalized stigma for people living with mental illness: a systematic review and meta-analysis. Soc Sci Med.

[16] Mak WWS, Cheung RYM (2010). Self-stigma among concealable minorities in Hong Kong: conceptualization and unified measurement. Am J Orthopsychiatry.

[17] Corrigan PW, Larson JE, Rüsch N (2009). Self-stigma and the "why try" effect: impact on life goals and evidence-based practices. World Psychiatry.

[18] Pinfold V, Thornicroft G, Huxley P, Farmer P (2005). Active ingredients in anti-stigma programmes in mental health. Int Rev Psychiatry.

[19] Corrigan PW, Morris SB, Michaels PJ, Rafacz JD, Rüsch N (2012). Challenging the public stigma of mental illness: a meta-analysis of outcome studies. Psychiatr Serv.

[20] Clement S, van Nieuwenhuizen A, Kassam A, Flach C, Lazarus A, de Castro M, McCrone P, Norman I, Thornicroft G (2012). Filmed v. live social contact interventions to reduce stigma: randomised controlled trial. Br J Psychiatry.

[21] Corrigan PW, Larson J, Sells M, Niessen N, Watson AC (2007). Will filmed presentations of education and contact diminish mental illness stigma?. Community Ment Health J.

[22] Kalyanaraman SS, Penn DL, Ivory JD, Judge A (2010). The virtual doppelganger: effects of a virtual reality simulator on perceptions of schizophrenia. J Nerv Ment Dis.

[23] Kilteni K, Groten R, Slater M (2012). The sense of embodiment in virtual reality. Presence Teleoperators Virtual Environments.

[24] Peck TC, Seinfeld S, Aglioti SM, Slater M (2013). Putting yourself in the skin of a black avatar reduces implicit racial bias. Conscious Cogn.

[25] Petkova VI, Khoshnevis M, Ehrsson HH (2011). The perspective matters! Multisensory integration in ego-centric reference frames determines full-body ownership. Front Psychol.

[26] Yee N, Bailenson JN (2006). Walk a mile in digital shoes: the impact of embodied perspective-taking on the reduction of negative stereotyping in immersive virtual environments.

[27] Ando S, Clement S, Barley EA, Thornicroft G (2011). The simulation of hallucinations to reduce the stigma of schizophrenia: a systematic review. Schizophr Res.

[28] Brown SA (2010). Implementing a brief hallucination simulation as a mental illness stigma reduction strategy. Community Ment Health J.

[29] Brown SA, Evans Y, Espenschade K, O'Connor M (2010). An examination of two brief stigma reduction strategies: filmed personal contact and hallucination simulations. Community Ment Health J.

[30] Cangas AJ, Navarro N, Parra JMA, Ojeda JJ, Cangas D, Piedra JA, Gallego J (2017). Stigma-Stop: a serious game against the stigma toward mental health in educational settings. Front Psychol.

[31] Fisher S, Cleveland SE (1968). Body Image and Personality. 2nd edition.

[32] Biocca F (1997). The cyborg's dilemma: progressive embodiment in virtual environments. J Computer-Mediated Commun.

[33] Maister L, Slater M, Sanchez-Vives MV, Tsakiris M (2015). Changing bodies changes minds: owning another body affects social cognition. Trends Cogn Sci.

[34] Green MC, Kass S, Carrey J, Herzig B, Feeney R, Sabini J (2008). Transportation across media: repeated exposure to print and film. Media Psychol.

[35] Tal-Or N, Cohen J (2010). Understanding audience involvement: conceptualizing and manipulating identification and transportation. Poetics.

[36] Green MC, Brock TC (2000). The role of transportation in the persuasiveness of public narratives. J Pers Soc Psychol.

[37] Caputo NM, Rouner D (2011). Narrative processing of entertainment media and mental illness stigma. Health Commun.

[38] Mann CE, Himelein MJ (2008). Putting the person back into psychopathology: an intervention to reduce mental illness stigma in the classroom. Soc Psychiatry Psychiatr Epidemiol.

[39] Oliver MB, Dillard JP, Bae K, Tamul DJ (2012). The effect of narrative news format on empathy for stigmatized groups. Journalism Mass Commun Q.

[40] Winterbottom A, Bekker HL, Conner M, Mooney A (2008). Does narrative information bias individual's decision making? A systematic review. Soc Sci Med.

[41] Kaufman GF, Libby LK (2012). Changing beliefs and behavior through experience-taking. J Pers Soc Psychol.

[42] Appel M, Richter T (2010). Transportation and need for affect in narrative persuasion: a mediated moderation model. Media Psychol.

[43] Green MC, Brock TC, Kaufman GF (2004). Understanding media enjoyment: the role of transportation into narrative worlds. Commun Theory.

[44] Schutte NS, Stilinović EJ (2017). Facilitating empathy through virtual reality. Motivation Emotion.

[45] Mak WWS, Chong ESK, Wong CCY (2014). Beyond attributions: understanding public stigma of mental illness with the common sense model. Am J Orthopsychiatry.

[46] Ehrsson HH (2007). The experimental induction of out-of-body experiences. Science.

[47] Slater M, Spanlang B, Sanchez-Vives MV, Blanke O (2010). First person experience of body transfer in virtual reality. PLoS One.

[48] Longo MR, Schüür F, Kammers MPM, Tsakiris M, Haggard P (2008). What is embodiment? A psychometric approach. Cognition.

[49] Hayes A (2013). Introduction to Mediation, Moderation, and Conditional Process Analysis: A Regression-Based Approach.

[50] Lysaker PH, Davis LW, Warman DM, Strasburger A, Beattie N (2007). Stigma, social function and symptoms in schizophrenia and schizoaffective disorder: associations across 6 months. Psychiatry Res.

[51] Sarge MA, Kim H, Velez JA (2020). An intervention: the role of perspective taking in combating public stigma with virtual simulations. Cyberpsychol Behav Soc Netw.

[52] Rohrer MW (2000). Seeing is believing: the importance of visualization in manufacturing simulation.

[53] Corrigan PW, Bink AB, Fokuo JK, Schmidt A (2015). The public stigma of mental illness means a difference between you and me. Psychiatry Res.

[54] Reinke RR, Corrigan PW, Leonhard C, Lundin RK, Kubiak MA (2004). Examining two aspects of contact on the stigma of mental illness. J Soc Clin Psychol.

[55] Thornicroft G, Rose D, Kassam A, Sartorius N (2007). Stigma: ignorance, prejudice or discrimination?. Br J Psychiatry.

[56] Gillath O, McCall C, Shaver PR, Blascovich J (2008). What can virtual reality teach us about prosocial tendencies in real and virtual environments?. Media Psychol.

